# Case Report: Complete remission after radiotherapy in two cases of locally advanced extramammary Paget’s disease of the perineum

**DOI:** 10.3389/fonc.2026.1801166

**Published:** 2026-04-13

**Authors:** Hui Ma, Xiaohui Xie, Xiaodong Peng

**Affiliations:** West China School of Medicine, Sichuan University, Sichuan University affiliated Chengdu Second People's Hospital, Chengdu Second People's Hospital, Chengdu, China

**Keywords:** cutaneous adenocarcinoma, extramammary Paget’s disease, locally advanced, non-surgical treatment, radiotherapy

## Abstract

**Purpose:**

Reports on the efficacy of radiotherapy for perineal extramammary Paget’s disease (EMPD) are limited. We present two cases of locally advanced perineal EMPD successfully treated with radiotherapy.

**Methods:**

A retrospective review was conducted on the clinical presentation, management, and outcomes of two patients with locally advanced, inoperable perineal EMPD who received radiotherapy at our hospital.

**Results:**

Both patients achieved complete clinical and radiological remission following radiotherapy. At the last follow-up in January 2026, neither patient showed any evidence of local recurrence or distant metastasis.

**Conclusions:**

Radiotherapy is an effective and well-tolerated definitive treatment modality for patients with locally advanced EMPD who are not candidates for or who decline surgical resection.

## Background

Extramammary Paget’s disease (EMPD) is a rare cutaneous adenocarcinoma. Its crude prevalence in mainland China was recently reported as 0.04 per 100,000 population (1). While more common in men within Asian populations, it typically presents in the elderly, with a mean age at diagnosis of 60–70 years (2). Wide local excision remains the standard primary treatment. However, surgery may be contraindicated due to advanced age, significant comorbidities, extensive disease precluding clear margins, or patient preference. For such inoperable cases, radiotherapy has emerged as an effective alternative, supported by retrospective data (3).

## Case presentations

### Case 1

**Figure 1:**
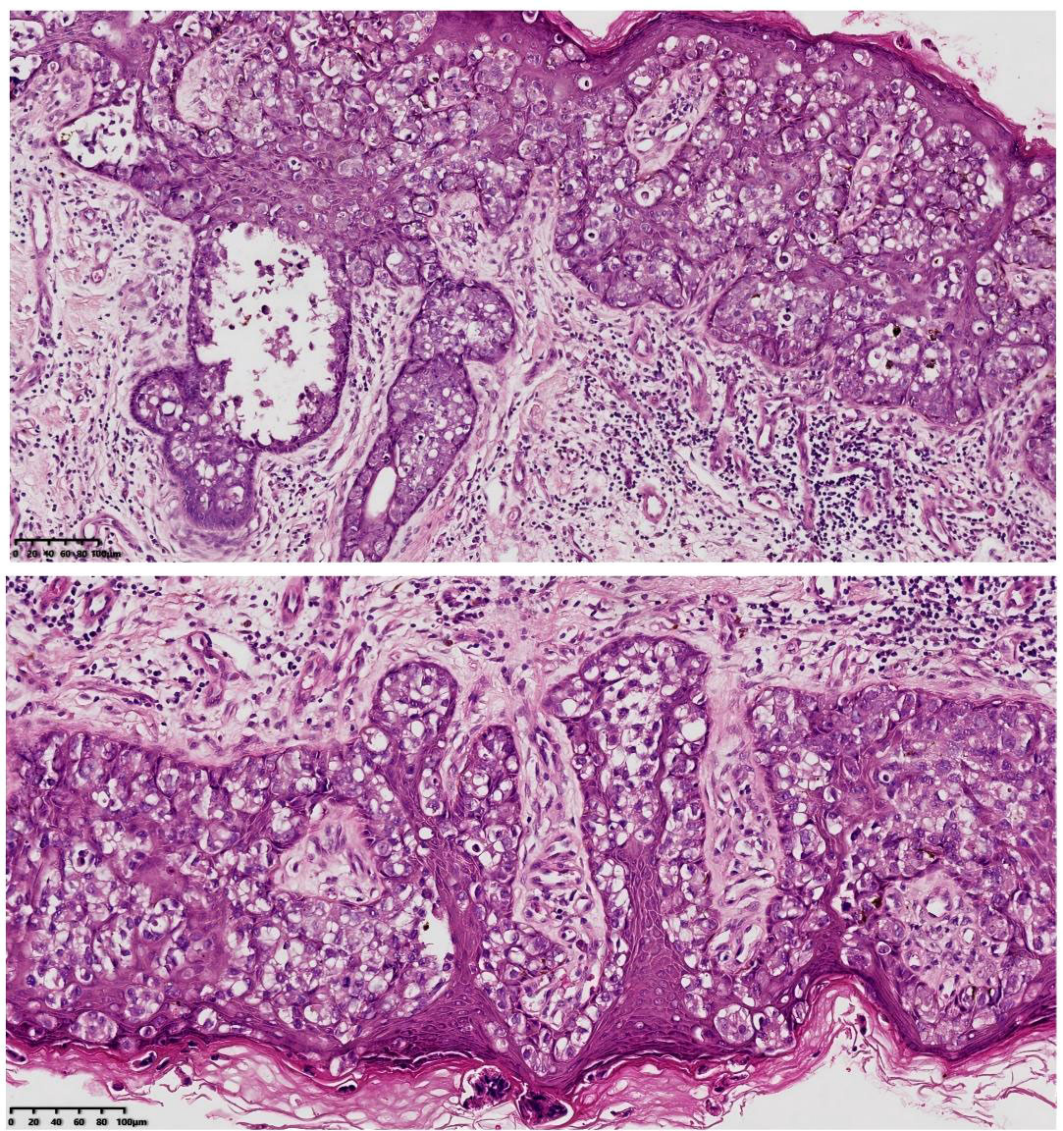
Pathological section (magnification ×200).

An 81-year-old man presented with a 3-year history of recurrent, pruritic erythematous plaques with scaling and erosion involving the perineum, scrotum, and penis. A history of intermittent pruritus in the same region spanned over a decade. Temporary improvement was noted with topical treatments, but lesions consistently recurred. A diagnostic skin biopsy in May 2022 confirmed EMPD (**Figure 1**). Immunohistochemical staining was positive for CK7, CEA, EMA, GCDFP-15, and HER2, and negative for S-100, p63, and CDX-2. The Ki-67 proliferation index was 45%. Staging imaging revealed no lymph node or distant metastases. A multidisciplinary evaluation deemed the patient a poor surgical candidate due to the extensive multifocal involvement, potential for significant functional morbidity, and the patient’s own preference against surgery. Consequently, definitive radiotherapy was recommended.

**Figure 2:**
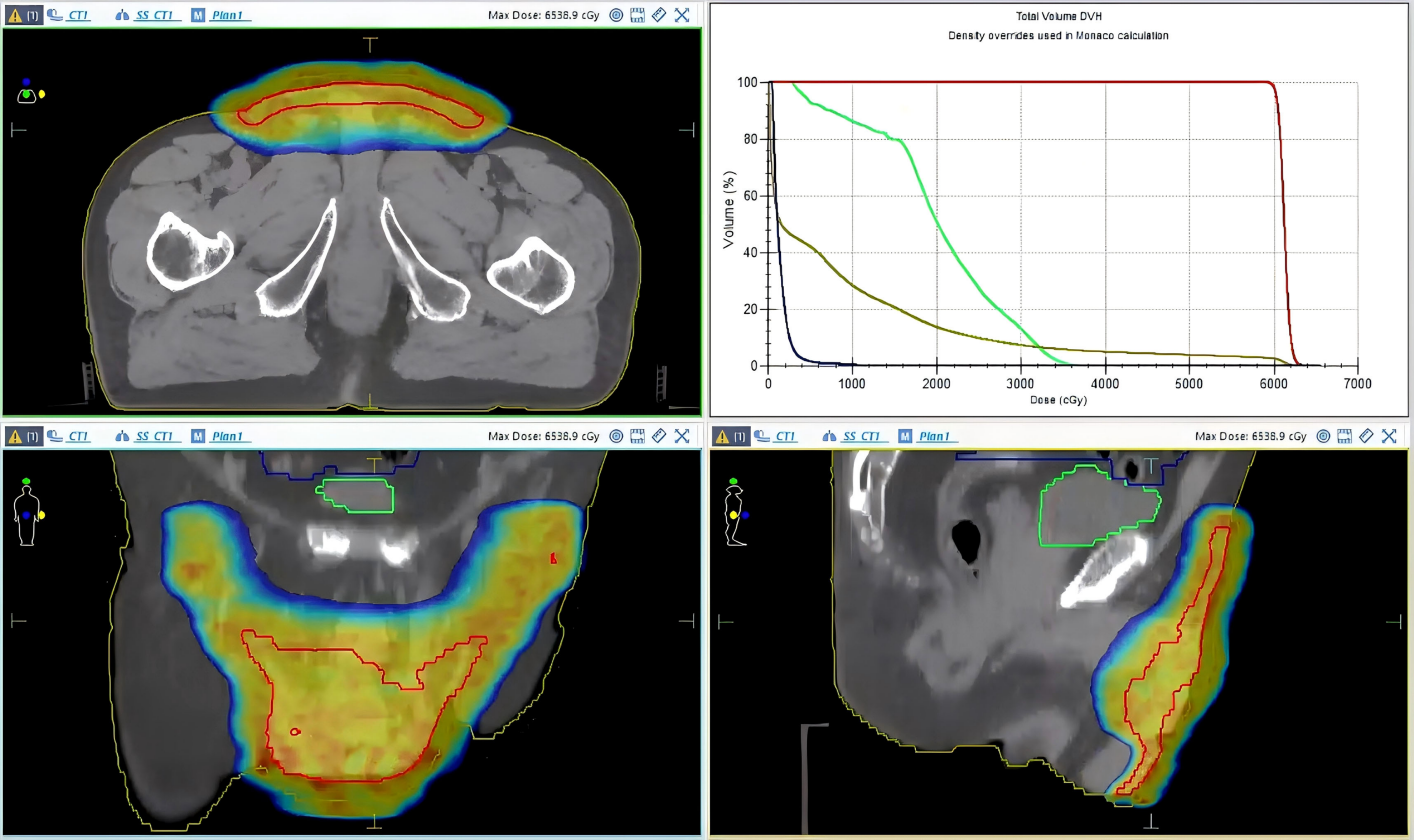
Treatment planning.

The patient underwent three-dimensional conformal intensity-modulated radiotherapy (IMRT) beginning in May 2022. The gross tumor volume (GTV) encompassed all visible lesions in the scrotum, penis, and perineum. The clinical target volume (CTV) included the GTV with a 1- to 2-cm margin. A total dose of 60 Gy was delivered in 30 fractions (2.0 Gy per fraction) over 6 weeks (**Figure 2**). A tissue-equivalent bolus was applied to the skin surface over the lesion to optimize the dose distribution and reduce skin side effects. Treatment was well-tolerated, with only Grade 1–2 radiation dermatitis and Grade 1 urinary symptoms reported. No hematologic or significant gastrointestinal toxicity occurred. Post-treatment evaluation confirmed complete clinical remission. The patient remained disease-free at the last follow-up in January 2026.

### Case 2

**Figure 3:**
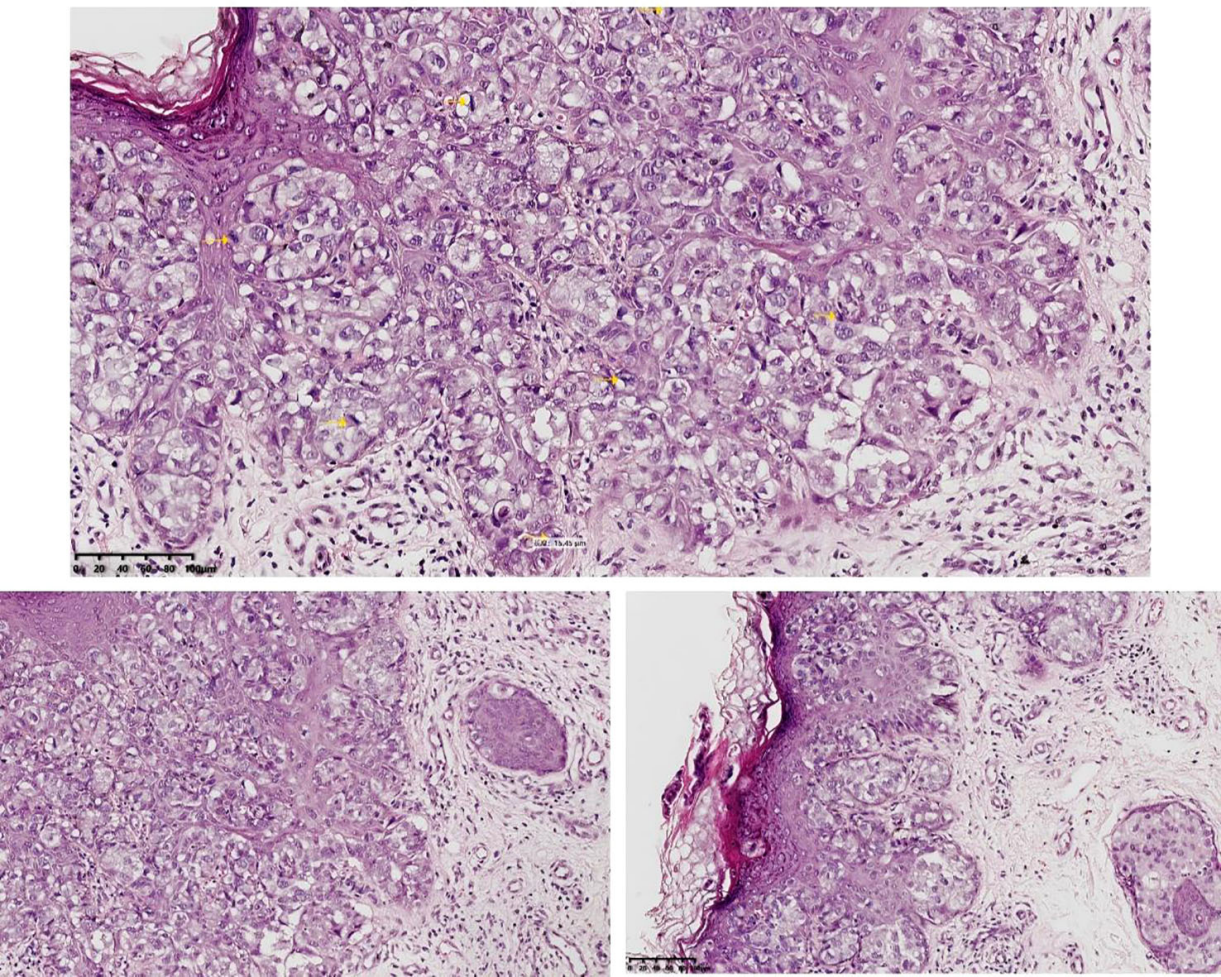
Pathological section (magnification ×200).

A 61-year-old man with a 5-year history of persistent, itchy erythema with scaling on the scrotum and penis was referred to our clinic. Pelvic magnetic resonance imaging demonstrated diffuse skin thickening of the penis, anterior lower abdominal wall, and left scrotum with nodular changes, highly suggestive of neoplastic involvement. Biopsy confirmed EMPD in November 2023 (**Figure 3**). Immunohistochemical findings were as follows: CK7 (+), CEA (+), GATA3 (+), GCDFP-15 (−), CK20 (−), P63 (−), CDX-2 (−), S-100 (−), Melan-A (−), CK5/6 (−), ER (−), HER2 (+), and Ki-67 (+, 40%). The patient’s medical history included type 2 diabetes. After multidisciplinary evaluation, radical surgery was deemed unsuitable, and radiotherapy was initiated in November 2023.

**Figure 4:**
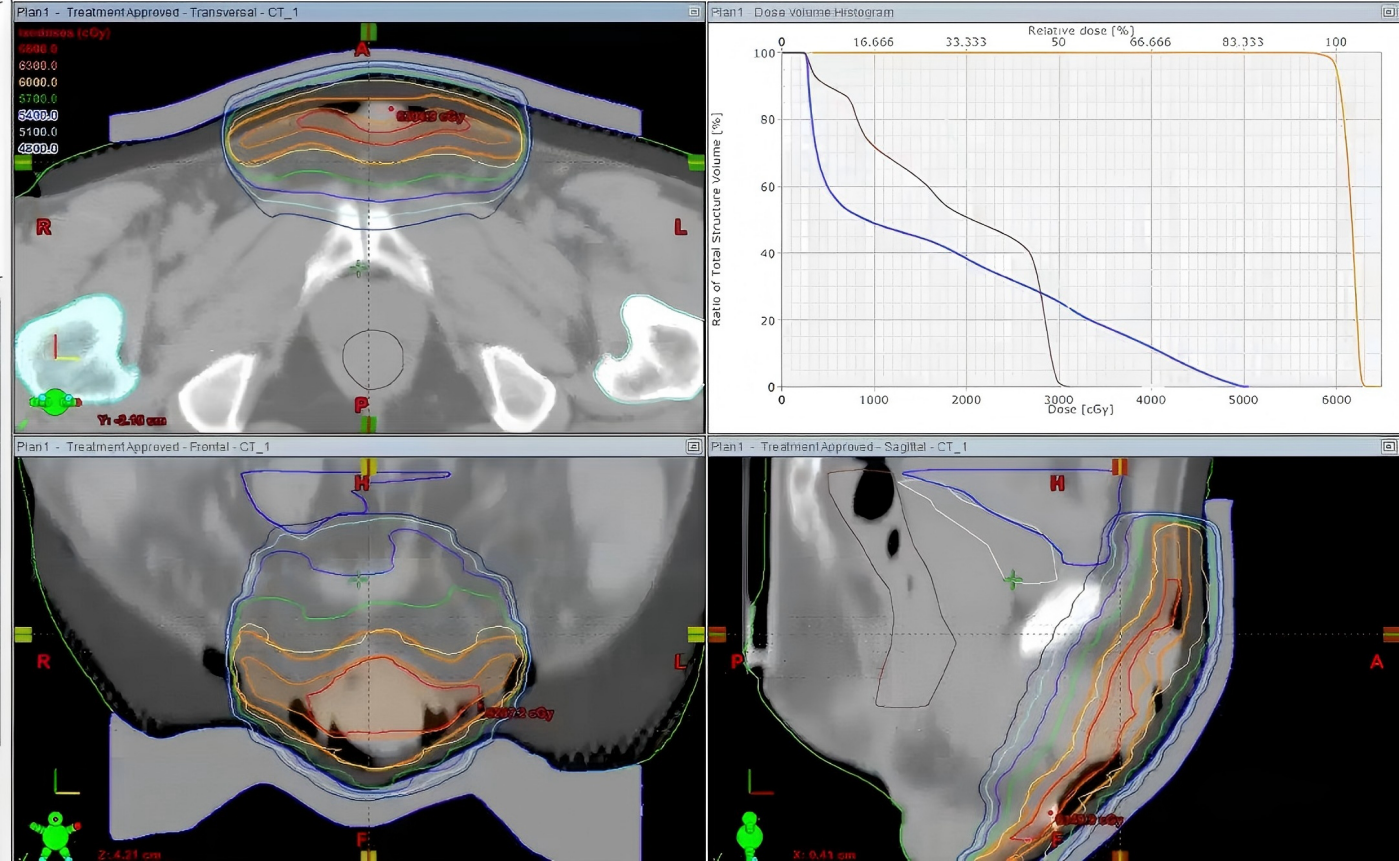
Treatment planning.

The patient received three-dimensional conformal radiotherapy targeting the involved scrotal, penile, and perineal skin, including adjacent high-risk areas. A total dose of 60 Gy was administered in 30 fractions (2.0 Gy per fraction) over 6 weeks (**Figure 4**). A bolus was placed on the lesion as part of the radiotherapy plan to reduce side effects. Treatment was completed without any Grade 3 or higher adverse events. The patient achieved a complete clinical response. Surveillance until January 2026 showed no evidence of recurrence or metastasis.

## Discussion

The management of EMPD, particularly in advanced or inoperable stages, lacks high-level evidence from prospective trials. Current strategies are derived from retrospective analyses and case series. While surgical resection is the cornerstone for localized disease, non-surgical modalities play a crucial role in specific clinical scenarios (3).

Radiotherapy has demonstrated significant efficacy as a primary treatment for EMPD. Hata et al. reported excellent local control (98% at 2 years) of metastatic lymph nodes from EMPD with radiotherapy, with a favorable toxicity profile (4). In a multi-institutional study, Niwa et al. observed a 95% objective response rate in patients with inoperable EMPD treated with radiotherapy, identifying lymph node metastasis as a key prognostic factor (5). A systematic review by Tagliaferri et al. concluded that radiotherapy offers high complete response rates (50%–100%) for both primary and recurrent EMPD, with predominantly mild (Grade ≤2) toxicities (6). Furthermore, a meta-analysis by Snast et al. found radiotherapy to have the highest complete response rate (97%) among non-surgical options, surpassing imiquimod (54%) and photodynamic therapy (36%) (7). These data support radiotherapy as a definitive and effective treatment for patients who are not candidates for surgery, as illustrated by the two cases presented here.

The optimal radiotherapy dose and volume for EMPD are not standardized. Reported total doses range from 45 to 80 Gy, with a median of 56–60 Gy commonly employed (6, 8, 9). Based on existing data, a dose of at least 60 Gy appears necessary for the lasting control of macroscopic disease (8). This aligns with recommendations from the Japanese Dermatological Association guidelines, which suggest radiotherapy as a curative option for patients unsuitable for surgery (10). Prognostic factors associated with poorer outcomes include dermal invasion and regional lymph node metastasis (8). In our practice, we utilized a CTV margin of 1–2 cm around gross disease, and no marginal recurrences were observed, aligning with recommendations from prior studies (8).

Looking forward, exploring combination therapies may further improve outcomes for inoperable EMPD. Rational strategies could include radiotherapy combined with systemic agents such as chemotherapy, anti-HER2 targeted therapy (particularly in HER2-positive cases), or immune checkpoint inhibitors. However, clinical data on such combinations remain sparse. More robust prospective research is warranted to define the optimal integration of radiotherapy with systemic therapy in the management of advanced EMPD.

## Conclusions

Based on our experience and the available literature, definitive radiotherapy appears to be a reasonable alternative for achieving local disease control in patients with locally advanced, inoperable EMPD. Further prospective studies are warranted to better define its optimal use.

## Data Availability

The original contributions presented in the study are included in the article/supplementary material. Further inquiries can be directed to the corresponding author.
